# Functional Repertoire of Antibiotic Resistance Genes in Antibiotic Manufacturing Effluents and Receiving Freshwater Sediments

**DOI:** 10.3389/fmicb.2017.02675

**Published:** 2018-01-17

**Authors:** Juan J. González-Plaza, Ana Šimatović, Milena Milaković, Ana Bielen, Fabienne Wichmann, Nikolina Udiković-Kolić

**Affiliations:** ^1^Division for Marine and Environmental Research, Ruđer Bošković Institute, Zagreb, Croatia; ^2^Division of Molecular Biology, Ruđer Bošković Institute, Zagreb, Croatia; ^3^Department of Biochemical Engineering, Faculty of Food Technology and Biotechnology, University of Zagreb, Zagreb, Croatia; ^4^Independent Researcher, Lausanne, Switzerland

**Keywords:** antibiotic resistance, effluent, manufacturing, antibiotic pollution, sediment, macrolides, functional metagenomics

## Abstract

Environments polluted by direct discharges of effluents from antibiotic manufacturing are important reservoirs for antibiotic resistance genes (ARGs), which could potentially be transferred to human pathogens. However, our knowledge about the identity and diversity of ARGs in such polluted environments remains limited. We applied functional metagenomics to explore the resistome of two Croatian antibiotic manufacturing effluents and sediments collected upstream of and at the effluent discharge sites. Metagenomic libraries built from an azithromycin-production site were screened for resistance to macrolide antibiotics, whereas the libraries from a site producing veterinary antibiotics were screened for resistance to sulfonamides, tetracyclines, trimethoprim, and beta-lactams. Functional analysis of eight libraries identified a total of 82 unique, often clinically relevant ARGs, which were frequently found in clusters and flanked by mobile genetic elements. The majority of macrolide resistance genes identified from matrices exposed to high levels of macrolides were similar to known genes encoding ribosomal protection proteins, macrolide phosphotransferases, and transporters. Potentially novel macrolide resistance genes included one most similar to a 23S rRNA methyltransferase from *Clostridium* and another, derived from upstream unpolluted sediment, to a GTPase HflX from *Emergencia*. In libraries deriving from sediments exposed to lower levels of veterinary antibiotics, we found 8 potentially novel ARGs, including dihydrofolate reductases and beta-lactamases from classes A, B, and D. In addition, we detected 7 potentially novel ARGs in upstream sediment, including thymidylate synthases, dihydrofolate reductases, and class D beta-lactamase. Taken together, in addition to finding known gene types, we report the discovery of novel and diverse ARGs in antibiotic-polluted industrial effluents and sediments, providing a qualitative basis for monitoring the dispersal of ARGs from environmental hotspots such as discharge sites of pharmaceutical effluents.

## Introduction

Antibiotic resistance is one of the most serious global public health threats of the twenty-first century (Carlet et al., 2012; ECDC, 2016; O'neill, 2016). This phenomenon is strongly associated with hospitals and other clinical environment (Brown et al., 2006; Rodriguez-Mozaz et al., 2015), because the extensive use of antibiotics in clinical settings is the driving force for increasing antibiotic resistance. However, there is a growing awareness that anthropogenic inputs of antibiotics into the environment through effluents, use of manure and biosolids in agriculture, and aquaculture contribute to this problem. The selection pressure imposed by antibiotics and other selective agents has promoted the propagation of antibiotic resistant bacteria (ARB) and antibiotic resistance genes (ARGs) (collectively known as the resistome) in the environment, creating vast reservoirs of ARGs with potential to be transferred to pathogens (Bengtsson-Palme et al., 2014; Cabello et al., 2016; Tao et al., 2016; Peng et al., 2017; Su et al., 2017). Understanding these reservoirs and behaviors of ARGs is crucial to control the emergence of resistant pathogens at a global scale.

Direct discharge of pharmaceutical effluents in receiving water bodies has been recognized as an important source of pollution, as they may contain high concentrations of antibiotics, ARB, heavy metals, and other hazardous materials (Babić et al., 2007; Larsson et al., 2007; Li et al., 2009; Sim et al., 2011; Larsson, 2014; Bielen et al., 2017). High concentrations of antibiotics which are above the minimum inhibitory concentrations could cause death of many susceptible environmental microorganisms and enrich those genetically adapted, while sub-inhibitory concentrations exert a selective pressure, which act as a moving force in horizontal dissemination of ARGs (Baker-Austin et al., 2006; Tacão et al., 2014; Di Cesare et al., 2016; Navon-Venezia et al., 2017). Therefore, environments impacted by discharges from manufacturing of antibiotics are high risk environments for antibiotic resistance selection and dissemination into human or animal pathogens. Hence, it is essential to understand the contribution of manufacturing sites to the environmental resistome in more detail.

Studies addressing the impact of effluent discharge from antibiotic production on the resistome of the receiving environment are still scarce and limited to Asian countries. Several studies using culture- and PCR-based methods have reported the presence of multidrug resistant bacteria (MDR) in rivers receiving effluents from antibiotic production (Li et al., 2009, 2010; Sidrach-Cardona et al., 2014; Lübbert et al., 2017). Although these methods provide valuable information, their major limitations are that the bacteria need to be culturable under laboratory conditions or screening is limited to known ARGs. Sequence-based metagenomics has enabled the exploration of the total DNA of a sample, providing a broad spectrum of known ARGs. This methodology has been used to study river and lake sediments highly polluted with antibiotics (mostly fluoroquinolones) from bulk drug production in India, revealing a high diversity and promotion of resistance genes to several classes of antibiotics as well as their mobilization elements (Kristiansson et al., 2011; Bengtsson-Palme et al., 2014). Despite these extremely valuable findings, metagenomic sequencing cannot identify potentially novel ARGs nor gives information on the expression of the ARGs (Mullany, 2014). Functional metagenomics, which consists of heterologous expression of metagenomic DNA in a surrogate host and activity-based screening, is a useful approach to identify different types of functional ARGs, both known and novel. Furthermore, the genes discovered by functional metagenomics are, by definition, candidates for horizontal transfer, as they must be functional in a heterologous host (Crofts et al., 2017). This method has been successfully applied in exploring the ARGs in different matrices including the human microbiome (Sommer et al., 2009), soil (Udikovic-Kolic et al., 2014), manure (Wichmann et al., 2014), activated sludge (Parsley et al., 2010), river (Amos et al., 2014), and ocean (Hatosy and Martiny, 2015).

Recently, our study on effluents from two Croatian pharmaceutical industries showed that they are contaminated with high levels of antibiotics and culturable ARB (Bielen et al., 2017). Treated effluent from an azithromycin (AZI)-synthesizing factory contained occasionally high, mg L^−1^ concentrations of macrolide antibiotics, which are critical for use in human medicine (WHO, 2017). On the other hand, partially treated effluent from a formulation industry contained a range of antibiotics (fluoroquinolones, trimethoprim, sulfonamides, and tetracyclines) from low to high μg L^−1^ concentrations (Bielen et al., 2017). These effluents are discharged into the nearby river and stream providing an opportunity for selection of antibiotic resistance. There is also the possibility that these environments harbor yet undescribed resistance genes, some of which we may face in pathogens in the hospitals tomorrow. Previous studies, applying metagenomic sequencing, have shown high levels of known and mobile ARGs, particularly those conferring resistance to quinolones and sulphonamides, in Indian sediments heavily polluted with fluoroquinolone antibiotics (Kristiansson et al., 2011; Bengtsson-Palme et al., 2014). In this study, using functional metagenomics we have explored the diversity of ARGs in industrial effluents and sediments polluted with macrolide or different veterinary antibiotics. We have also assessed the genetic context of the identified resistance genes by analyzing their flanking DNA.

## Materials and methods

### Study areas and sampling procedures

Study areas lie in the northwest of Croatia where two local pharmaceutical industries are situated. Industry 1 has a long tradition in synthesizing the macrolide antibiotic AZI and discharges its final, treated effluent into the Sava river. Industry 2 formulates various veterinary antibiotics, including antibiotics from the tetracycline, sulfonamide and beta-lactam classes, and discharges its partially treated effluent into the nearby small stream. More detailed information about these two industries and properties of their effluents have been recently published (Bielen et al., 2017).

Industrial effluents and sediment samples from the receiving river and stream were collected in January and February 2016. Effluent from Industry 1 was collected as grab sample from the discharge pipe and effluent from Industry 2 consisted of a 24-h composite sample. Both effluent samples were collected in a sterile 2 L bottle and kept at 4°C. Immediately upon return to the laboratory, aliquots of 50–100 mL were vacuum-filtered through mixed cellulose ester filters (0.22 μm pore diameter) (GE Healthcare Life Sciences) to collect the bacterial cells, and filters were stored at −80°C until DNA extraction. Surface sediment samples (0–10 cm) were taken at effluent discharge locations and at reference locations situated upstream of the discharge areas (4 samples in total). Four sub-samples were collected at each location and merged to a composite sample (10 g of each sub-sample) within 4 h of collection. These samples were used fresh for immediate culturing or stored at −80°C until DNA was extracted.

### Culturing bacteria from sediments

To culture bacteria from fresh sediment samples, 1 g of the composite sediment was suspended in physiological saline (0.9% NaCl) by vortexing. Serial 10-fold dilutions were cultured on three replicate R2A agar plates to enumerate total bacteria. To enumerate resistant bacteria, serial dilutions were cultured in triplicates on plates supplemented with AZI (15 mg L^−1^) (Fluka, Germany) for Industry area 1 samples; or sulfamethazine (SMZ; 350 mg L^−1^) (Sigma, Germany) or oxytetracycline (OTC; 25 mg L^−1^) (Sigma, Germany) for Industry area 2 samples. Colony forming units (CFU) were counted after a 5 day incubation at 28°C. ARB cultured from sediments from discharge locations were scraped from the plates, pooled and stored in R2A broth containing antibiotic and 15% glycerol at −80°C.

### Small insert-size metagenomic library construction

DNA for the construction of libraries was isolated from the filters, sediment samples and from pools of cultured sediment bacteria using the PowerSoil DNA isolation kit (MoBio Laboratories, Carlsbad, CA) according to the manufacturer's recommendations. DNA was either partially digested with *Pst*I (NEB, USA) and cloned into the pCF430 vector (Newman and Fuqua, 1999) or digested with *Hind*III (NEB, USA) and cloned into the pZE21-MCS vector (Lutz and Bujard, 1997; Table 1). Ligation products were dialyzed using 0.2-μm filter membranes (Millipore, Ireland) and electroporated into *Escherichia coli* DH5α cells (Invitrogen, Carlsbad, CA) using a Micropulser (Biorad, Hercules, CA). After a 1 h incubation in SOC media, cells were plated on LB plates supplemented with 5 mg L^−1^ tetracycline (Industry area 1) or 50 mg L^−1^ kanamycin (Industry area 2), and incubated at 37°C overnight. Library storage and size estimation were performed according to previously published protocols (Wichmann et al., 2014). Briefly, the average insert size for each library was determined by restriction digest analysis of 10 randomly picked clones using *Pst*I (pCF430) or *Hind*III (pZE21-MCS). After insert size analysis, all clones were pooled together by scraping them from plates into LB supplemented with 20% glycerol and tetracycline or kanamycin followed by storage at −80°C.

**Table 1:** Features of the metagenomic libraries constructed in this study.

**Study area**	**Library name**	**Origin**	**Vector/AR marker**	**Average insert size (kb)**	**Amount of cloned DNA (Gb)**
Industry area 1	S_US_C1	Sediment at upstream site	pCF430/Tet^R^	4.40	5.47
	F_WW_C1	Pharmaceutical effluent	pCF430/Tet^R^	4.20	19.20
	B_DS_C1	Culturable AZI-resistant bacteria from sediment at discharge site	pCF430/Tet^R^	3.40	5.50
	S_DS_C1	Sediment at discharge site	pCF430/Tet^R^	2.80	1.54
Industry area 2	S_US_C2	Sediment at upstream site	pZE21-MCS/Kan^R^	3.70	2.20
	F_WW_C2	Pharmaceutical effluent	pZE21-MCS/Kan^R^	3.38	4.56
	B_DS_C2	Culturable SMZ and OTC-resistant bacteria from sediment at discharge site	pZE21-MCS/Kan^R^	2.90	1.96
	S_DS_C2	Sediment at discharge site	pZE21-MCS/Kan^R^	2.50	4.58

### Identification of antibiotic resistance genes

Metagenomic libraries (10 μL of the pooled clones) were grown in 5 mL of LB supplemented with the appropriate antibiotic (either tetracycline or kanamycin) for 2 h at 37°C and 200 rpm. Appropriate dilutions were plated on LB plates containing an antibiotic of interest: AZI (16 mg L^−1^) or erythromycin (ERI; 100 mg L^−1^) for Industry area 1 libraries; trimethoprim (TRM; 20 mg L^−1^), tetracycline (TET; 20 mg L^−1^), OTC (20 mg L^−1^) ampicillin (AMP; 100 mg L^−1^), cefotaxime (CTX; 8 mg L^−1^), or ciprofloxacin (1 and 0.5 mg L^−1^) for Industry area 2 libraries, and incubated overnight at 37°C. For screening of libraries from Industry area 2 on SMZ (350 mg L^−1^) and TRM, instead of LB media, Mueller-Hinton broth or agar was used because it is low in sulfonamide and trimethoprim inhibitors. The antibiotic concentrations used inhibited growth of *E. coli* DH5α transformed with empty pCF430 or pZE21-MCS plasmid. The proportion of resistant clones in each library was calculated as the ratio of the number of resistant clones (grown on plates containing an antibiotic of interest) and the total number of clones (grown on plates containing vector antibiotic). The diversity of individual resistant clones was assessed with *Hind*III and *Bam*HI digestion (libraries derived from Industry area 1) or with *Pst*I and *Bam*HI digestion (libraries derived from Industry area 2). Plasmids containing inserts with distinct restriction patterns were sent to Macrogen DNA Sequencing Service (Macrogen, Netherlands) for bi-directional Sanger sequencing using vector-targeting forward and reverse primers (Sommer et al., 2009; Wichmann et al., 2014). Additional specific primers were designed as necessary for extension of the obtained sequences. Sequencing data was processed using the DNASTAR Lasergene software package (version 14) and nucleotide sequences of the identified open reading frames (ORFs) were compared to the publicly available sequences using BLASTX search against the NCBI nr/nt database. Active gene was considered to be unique if it did not have identical nucleotide sequence to any other gene in the same library.

### Determination of minimum inhibitory concentration (MIC)

MIC assays were performed on unique resistant clones by broth microdilution in Mueller-Hinton broth (Difco, USA) according to previously published protocols (Donato et al., 2010). The MIC was defined as the lowest concentration of the antibiotic that inhibited visible growth of 10^5^ cells of tested clone. As a control we used DH5α cells transformed with the empty vector (pCF430 or pZE21-MCS).

### Phylogenetic analysis

The Geneious software (version 6.0.5) (Kearse et al., 2012) was used for sequence comparisons and phylogenetic analyses. For sequence alignments, we used CLUSTALW (Thompson et al., 2002), and the phylogenetic trees were inferred using maximum likelihood (Jones et al., 1992). Bootstrap values were calculated based on 100 replications. Trees were adapted using the FigTree program (v1.4.3.) (Rambaut, 2009).

### Nucleotide sequence accession numbers

The metagenomic insert sequences from Industry area 1 are shown in Table 2 and were deposited in GenBank under accession numbers MG585943 to MG585960. Sequences from Industry area 2 are shown in Supplementary Table 2 and were deposited under accession numbers MG585961 to MG586044.

**Table 2:** Summary of all macrolide resistance genes from clones with distinct restriction digest patterns in functional metagenomic libraries built from effluent and sediments of Industry area 1.

**Antibiotic used for selection**	**Clone designation/origin**	**MIC (mg L^−1^)**	**Gene length (bp)**	**Gene annotation (Closest BLASTX hit in NCBI)**	**% amino acid identity**	**GenBank Accession No**.
Azithromycin	AZI1_S_US_C1/ Upstream sediment	<16 (AZI) 128 (ERI)	1,281	GTPase binding protein HflX (*Emergencia timonensis* WP_067543614.1)^*^	63	MG585943
	AZI2_S_US_C1/ Upstream sediment	<16 (AZI) 128 (ERI)	1,281	GTPase binding protein HflX (*Emergencia timonensis* WP_067543614.1)^*^	63	MG585944
	AZI1_F_WW_C1/ Effluent	64 (AZI) 512 (ERI)	1,476	ABC-F type ribosomal protection protein Msr(E) (*Klebsiella pneumoniae* YP_003754030.1)^*^	100	MG585948
	AZI4_F_WW_C1/ Effluent	32 (AZI) 512 (ERI)	1,476	ABC-F type ribosomal protection protein Msr(E) (*Klebsiella pneumoniae* YP_003754030.1)^*^	99	MG585949
	AZI1_B_DS_C1/ Sediment bacteria	512 (AZI) 2,048 (ERI)	1,476	ABC-F type ribosomal protection protein Msr(E) (*Klebsiella pneumoniae* YP_003754030.1)^*^	100	MG585952
			885	Macrolide 2′-phosphotransferase Mph(E) (*Klebsiella pneumoniae* YP_003754029.1)^*^	100	MG585954
	AZI4_B_DS_C1/ Sediment bacteria	512 (AZI) 2,048 (ERI)	348	SMR family, quaternary ammonium compound efflux QacEΔ1 (*Salmonella enterica* NP_511227.1)^*^	100	MG585956
	AZI1_S_DS_C1/ Discharge sediment	256 (AZI) 1,024 (ERI)	1,476	ABC-F type ribosomal protection protein Msr(E) (*Acinetobacter baumannii* YP_724476.1)^*^	99	MG585957
Erythromycin	ERI1_S_US_C1/ Upstream sediment	64 (ERI) <16 (AZI)	1,281	GTPase binding protein HflX (*Emergencia timonensis* WP_067543614.1)	63	MG585945
	ERI2_S_US_C1/ Upstream sediment	128 (ERI) <16 (AZI)	1,278	GTPase binding protein HflX (*Emergencia timonensis* WP_067543614.1)^*^	62	MG585946
	ERI9_S_US_C1/ Upstream sediment	64 (ERI) <16 (AZI)	1,281	GTPase binding protein HflX (*Emergencia timonensis* WP_067543614.1)^*^	62	MG585947
	ERI1_F_WW_C1/ Effluent	512 (ERI) 32 (AZI)	1,224	MFS macrolide efflux protein Mef(C) (*Colwellia chukchiensis* WP_085286200.1)^*^	100	MG585951
	ERI4_F_WW_C1/ Effluent	1,024 (ERI) 64 (AZI)	1,473	ABC-F type ribosomal protection protein Msr(E) (*Klebsiella pneumoniae* YP_003754030.1)^*^	100	MG585950
	ERI2_B_DS_C1/ Sediment bacteria	1,024 (ERI) 256 (AZI)	1,476	ABC-F type ribosomal protection protein Msr(E) (*Acinetobacter baumannii* YP_724476.1)	100	MG585953
	ERI9_B_DS_C1/ Sediment bacteria	1,536 (ERI) <16 (AZI)	903	23S ribosomal RNA methyltransferase (*Clostridium* sp. CAG:780 CCZ18576.1)^*^	67	MG585955
	ERI1_S_DS_C1/ Discharge sediment	1,024 (ERI) 128 (AZI)	885	Macrolide 2'-phosphotransferase Mph(E) (*Acinetobacter baumannii* YP_001736317.1)^*^	100	MG585958
	ERI2_S_DS_C1/ Discharge sediment	1,024 (ERI) 64 (AZI)	1,224	MFS macrolide efflux protein Mef(C) (*Colwellia chukchiensis* WP_085286200.1)^*^	100	MG585959
	ERI7_S_DS_C1/ Discharge sediment	512 (ERI) <16 (AZI)	885	Macrolide 2′-phosphotransferase Mph(G) (*Colwellia chukchiensis* SEL95196.1)^*^	100	MG585960

Unique genes (based on their nucleotide sequence) from the same library are marked with ^*^. MIC, Minimal inhibitory concentration.

## Results and discussion

### Selection of antibiotic resistance in river and stream sediments receiving effluents from antibiotic manufacturing

**Figure 1:**
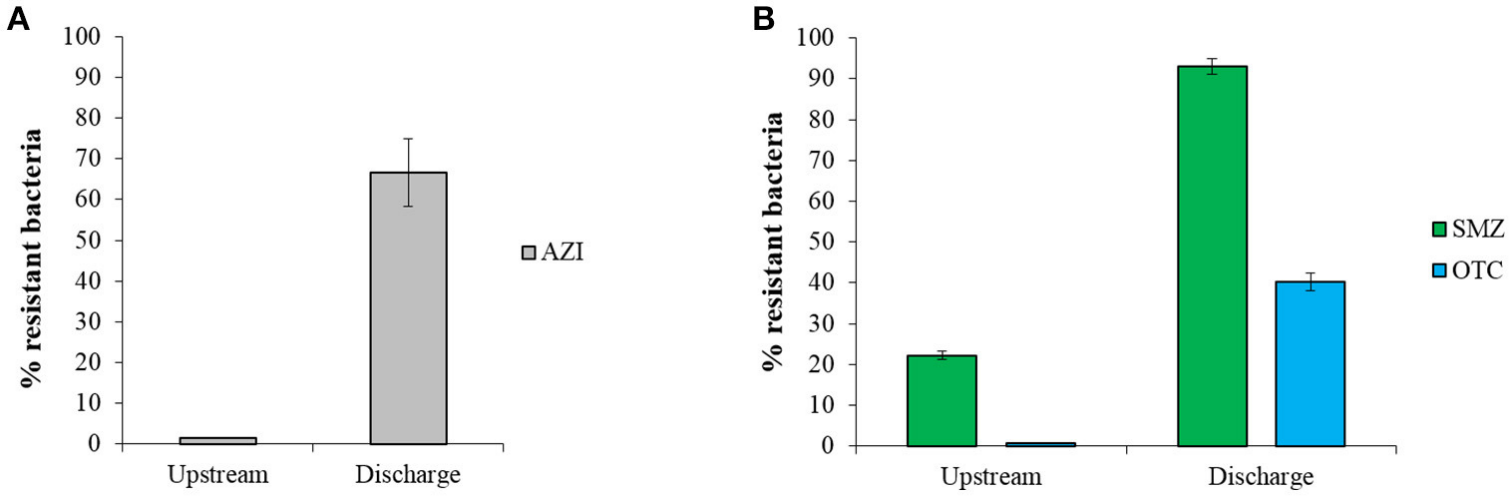
The proportion of resistance (mean ± SD) of culturable bacteria to **(A)** azithromycin (AZI) and **(B)** sulfamethazine (SMZ) and oxytetracycline (OTC) in sediments from discharge and upstream sites of two study areas. The percentage of antibiotic resistant bacteria was calculated as the ratio of resistant bacteria CFU and total CFU.

Culturing of sediment bacteria showed a considerably higher proportion of AZI-resistant or SMZ- and OTC-resistant bacteria in sediments at the discharge vs. upstream sites, indicating an enrichment of resistant populations in the effluent-impacted environment (Figure 1). Higher proportion of ARB has also been observed in the Indian sediment samples in comparison with samples from reference locations, most likely caused by the emissions of high concentrations of antibiotics from local drug manufacturers (Flach et al., 2015). In a recent study, we showed that effluents from two manufacturing sites studied here contained high concentrations of antibiotics and a high proportion of culturable ARB (Bielen et al., 2017). For example, mg L^−1^ levels of macrolide antibiotics along with high frequencies of AZI-resistant bacteria (up to 83%) were found in effluents from Industry area 1. Furthermore, effluents from Industry area 2 were found to contain high levels of SMZ- and OTC-resistant bacteria (up to 90 and 50%, respectively) and several antibiotics including sulfonamides, fluoroquinolones, trimethoprim and tetracyclines in concentrations up to about 230 μg L^−1^. Consequently, it would be reasonable to expect higher levels of these antibiotics in sediments at the discharge compared with upstream sites, as shown in our preliminary analyses (unpublished data). Therefore, the observed higher proportion of culturable ARB at both discharge sites could be due to a pollution of the river and stream with antibiotics that select for resistant bacteria already resident in the sediment. Alternatively, the released effluent-associated resistant bacteria may proliferate in the sediment, or a combination of these contributors may take place. Receiving sediments may thus act as a reservoir where known circulating resistant bacteria and their genes are maintained as well as new resistant strains and genes may emerge and spread.

Metagenomic DNA extracted from sediment and effluent samples was used to build eight small-insert libraries (2 from effluents, 2 from upstream sediments, and 4 from discharge sediments, Table 1). The libraries from Industry area 1 had an average insert size of 3.7 kb and an average total size of 32 Gb. The libraries from Industry area 2 contained a total of 13 Gb with an average insert size of 3.1 kb. The proportion of resistant clones selected on eight antibiotics was generally lower in libraries originating from upstream sediments compared with libraries from discharge sediments and effluents (Supplementary Figure 1), suggesting a selection of resistance genes in matrices impacted by pollution with antibiotics and ARB.

### Identification of macrolide resistance genes in effluent and river sediments near industry area 1

Screening of the libraries of Industry area 1 yielded 17 different macrolide resistant clones with 18 resistance genes, 16 of which were unique based on their nucleotide sequence (Table 2, Supplementary Table 2). These genes represented one unknown resistance mechanism mediated by a GTPase binding protein and three known mechanisms, such as efflux pumps, macrolide inactivation by phosphotransferases and target modification/protection by ribosomal RNA methyltransferases or ribosomal protection proteins (Table 2). To classify our annotated genes as novel genes, we set a cut-off of 80% protein sequence identity (Zhao et al., 2014).

The majority of unique genes (12/16) matched known ARGs encoding macrolide efflux pumps, ribosomal protection proteins and macrolide 2′-phosphotransferases (Supplementary Figure 2, Supplementary Table 1A). Efflux pump genes included two major facilitator superfamily (MFS) transporter genes with high sequence identity to *mef* (C) from *Colwellia chuchiensis* and one small multidrug resistance (SMR) transporter, highly similar to the quaternary ammonium compound efflux gene (*qacE*Δ*1*) from *Salmonella enterica*. The *mef* genes have largely been found among clinically relevant macrolide resistant pathogens (Fyfe et al., 2016). Ribosomal protection proteins were encoded by six ABC-F protein genes with high similarity to *msr*(E) previously found in *Klebsiella pneumoniae* and *Acinetobacter baumannii*. Recently, Sharkey et al. (2016) provided strong evidence that these proteins interact with the ribosome and displace the drug from its binding site, thus revealing a novel role for ABC-F proteins in antibiotic resistance. Macrolide inactivating enzymes included phosphotransferase genes, *mph*(E) and *mph*(G) which were identical (100% of amino acid identity) to those from *A. baumanii, K. pneumoniae*, and *C. chuchiensis*, respectively. All of these known genes (*mef, msr, mph*) were obtained from libraries deriving from antibiotic-impacted matrices (effluent and receiving sediment), suggesting their relation to selection pressure from macrolide antibiotics and possibly other co-selecting agents from AZI production.

Only one gene (*erm*, clone ERI9_B_DS_C1) deriving from the library from the discharge site shared low amino acid sequence identity (67%) with a 23S rRNA methyltransferase from *Clostridium* sp. (Table 2). This suggests that it encodes for a potentially novel member of this methyltransferase family, which confers clinically relevant levels of ERI resistance (MIC = 1,536 mg L^−1^; Table 2) through ribosomal methylation. The mechanism mediated by *erm* genes remains the most widespread mechanism of macrolide resistance in clinically important pathogens (Fyfe et al., 2016).

Contrary to most known genes in polluted matrices, all unique genes deriving from upstream reference sediment (4/16) had ≤63% protein sequence identity with their best hit in the NCBI, a GTPase HflX from *Emergencia timonensis*, indicating potential novelty of these genes (Table 2, Supplementary Table 1A, Supplementary Figure 2). Although the exact mechanism mediated by HflX remains to be unraveled, Lau et al. (2017) proposed that the GTPase HflX acts as an alternative ribosome splitting factor which disassembles the 70S ribosomes into its subunits and in this way helps to overcome the translational arrest caused by macrolides.

Based on MIC results, the HflX-mediated macrolide resistance seems less effective than the other identified mechanisms, which conferred a higher level of macrolide resistance (ERI 64–128 vs. 512–2,048 mg L^−1^; AZI 16 vs. 32–512 mg L^−1^, Table 2). This indicates that the bacteria living in sediment at the discharge site have evolved or acquired increasing resistance to macrolides in response to exposure to high macrolide selection pressure. In contrast, sediment bacteria from the upstream site that has had no known anthropogenic exposure to antibiotics are source of different, yet unknown mechanisms of macrolide resistance. These might evolve coincidentally in the presence of selective forces other than antibiotics that may cause accumulation of mutations that incidentally also confer antibiotic resistance (Knöppel et al., 2017). Other studies also showed that environments (i.e., sediments) not subjected to anthropogenic antibiotic pollution could be reservoirs of novel ARGs (Kristiansson et al., 2011; Amos et al., 2014; Nesme et al., 2014).

In addition, the discovery of potentially novel macrolide resistance genes originating from *Emergencia* and *Clostridium* genera in *E. coli* further demonstrates the power of functional metagenomics to identify resistance genes from Gram-positive bacteria in Gram-negative host.

### Identification and phylogeny of sulfonamide, tetracycline, trimethoprim, and beta-lactam resistance genes in effluent and environment near industry area 2

Of 82 clones with 84 genes conferring resistance to sulfonamides, tetracyclines, trimethoprim and beta-lactams, we obtained 66 unique ARGs (Supplementary Tables 1B, 2). No clones resistant to fluoroquinolones (ciprofloxacin) were obtained in this study, as has also been reported in one other study of soil (Charles et al., 2017), which may be due to incompatibility resulting from the use of *E. coli* as surrogate host. The predicted protein sequences of identified ARGs shared between 49 and 100% amino acid sequence identity with proteins in the NCBI database (Supplementary Figure 3), although the average sequence identity differed among the types of ARGs. For example, all of the genes conferring resistance to SMZ, OTC, and TET were highly similar (amino acid identity ≥94%) with previously known genes. SMZ resistant clones contained the dihydropteroate synthase genes (*sul1* and *sul2*), which are also found in pathogens such as *Enterobacter cloacae* and *A. baumannii* (Supplementary Table 2). The fact that the *sul1* gene was detected in all of the four libraries suggests a wide distribution of this gene in background sediment and industrial effluent. In contrast, the *sul2* was detected in sediment only at the discharge site, suggesting that its presence may have resulted from effluent discharge. However, both genes (*sul1* and *sul2*) have been previously reported in antibiotic polluted (Luo et al., 2010; Kristiansson et al., 2011; Bengtsson-Palme et al., 2014) and unpolluted sediments (Czekalski et al., 2015; Archundia et al., 2017), which is likely due to their genetic localization on mobile elements that could be easily transferred among bacteria (Heuer et al., 2011; Hu et al., 2016; Johnson et al., 2016; Koczura et al., 2016).

All functional genes from OTC and TET resistant clones matched previously reported tetracycline transporters that belong to the MFS, indicating that efflux is the predominant mechanism of resistance to tetracyclines in Industry area 2 (Supplementary Table 2). Phylogenetic analysis (Supplementary Figure 4) showed that the majority of tetracycline and oxytetracycline resistance genes (14/21), identified from all four libraries, cluster closely together with a *tet* transporter gene from *Flavobacterium psychrophilum* which is not similar to any annotated group of *tet* resistance genes. The detection of these genes in all four libraries suggests their natural distribution in the sediment and industrial effluent. The rest of the sequences are related to *tet*(39), *tet*(A), or *tet*(C) from the γ-*Proteobacteria*. These genes have also been found in a highly antibiotic polluted lake sediment, with the *tet*(39) being the most abundant (Bengtsson-Palme et al., 2014). Of these genes, we only detected *tet*(C) in our upstream sediment, suggesting that it may occur naturally in the studied sediment or it comes from nearby agricultural sources. However, other studies detected *tet* resistance genes, including *tet*(A) and *tet*(C), in environments not subjected to anthropogenic antibiotic pollution (Andersen and Sandaa, 1994; West et al., 2011; Durso et al., 2016), implying their wide distribution in the environment.

**Figure 2:**
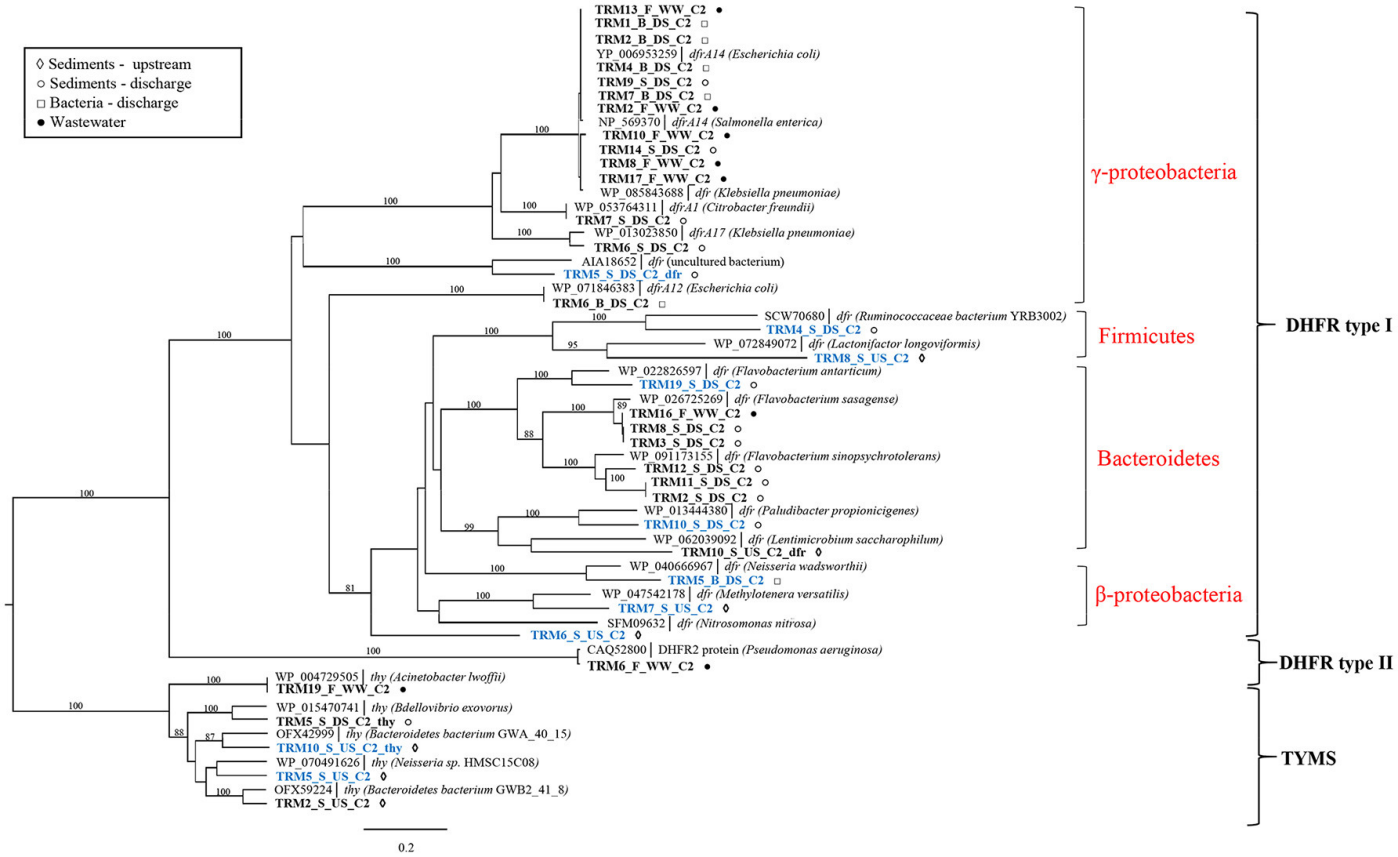
Phylogenetic tree of protein sequences of TRM resistance genes. Best BLAST hits and representative protein sequences of the studied genes were retrieved from the NCBI database. The evolutionary history was inferred by using the maximum likelihood method and the Geneious software. Bootstrap values were calculated on 100 replications and only those higher than 80% are shown. Sequences that share ≤80% amino acid identity with proteins in the NCBI database are shown in blue. Scale bar = 0.2 changes/site.

Screening of libraries with TRM resulted in a substantial proportion (11/35) of potentially novel genes (≤80% amino acid identity) along with the known genes (Supplementary Table 2). Both known and all potentially novel genes were found in libraries of both upstream and discharge sediment indicating that sediment itself is a natural reservoir for TRM-resistant bacteria carrying a diverse set of known and unknown TRM resistance genes (Supplementary Table 2). All identified genes were predicted to encode target-modified dihydrofolate reductases (DHFR) or thymidylate synthases (TYMS). Phylogenetic clustering showed that the majority of identified genes are distributed in the cluster containing type I DHFRs from the *Proteobacteria, Firmicutes*, and *Bacteroidetes* (Figure 2). Within this cluster, known resistance genes were mainly related to *dfr14, dfrA1*, and *dfr17* genes from pathogenic γ*-Proteobacteria* or *dfr* from *Bacteroidetes*. In contrast, novel resistance genes formed a separate clusters with DHFR type I proteins from β*-Proteobacteria, Firmicutes*, and *Bacteroidetes*. Only one gene (TRM6_F_WW_C2) originating from effluent was identified as type II DHFR gene from *Pseudomonas aeruginosa* (99% homology). In addition, the TYMS group of sequences included both known and novel *thy* genes found in the *Proteobacteria* and *Bacteroidetes*.

**Figure 3:**
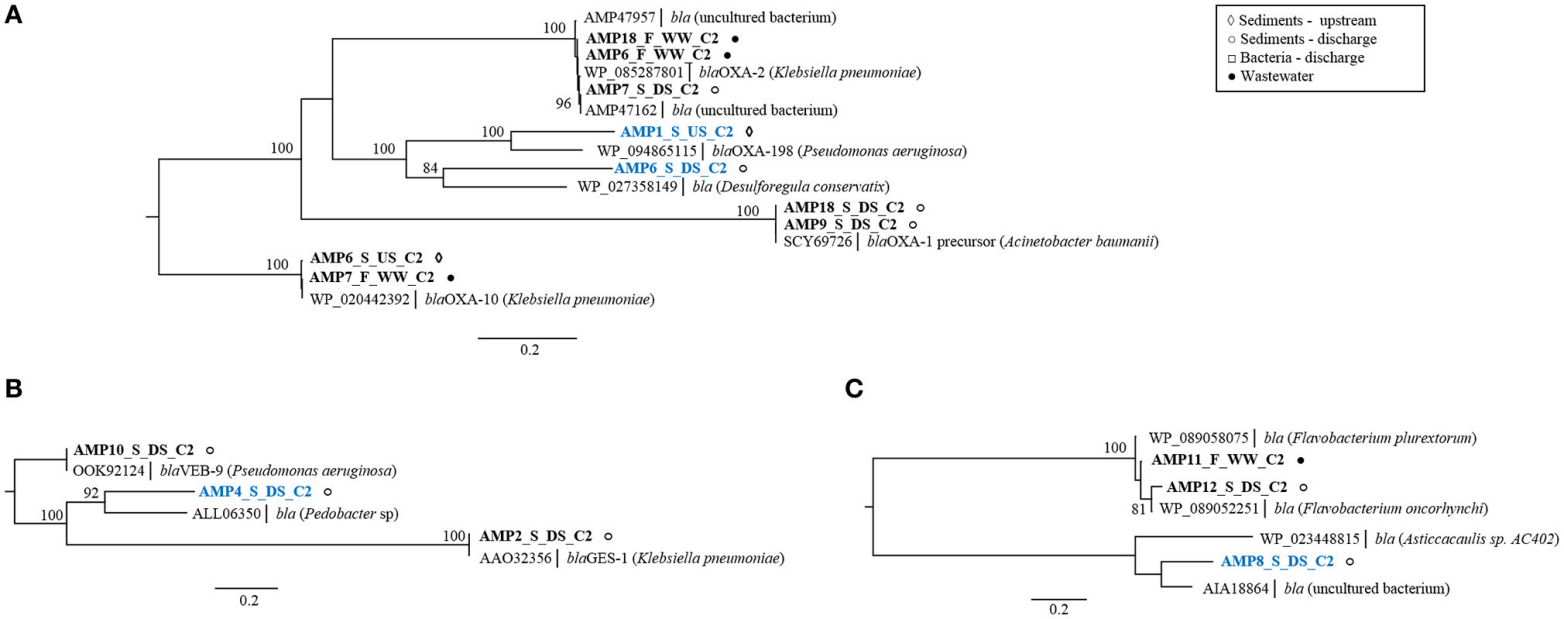
Phylogenetic trees of protein sequences of class D beta-lactamases **(A)**, class A beta-lactamases **(B)**, and class B beta-lactamases **(C)**. Best BLAST hits and representative protein sequences of the studied gene were retrieved from the NCBI database. The evolutionary history was inferred by using the maximum likelihood method and the Geneious software. Bootstrap values were calculated on 100 replications and only those higher than 80% are shown. Sequences that share ≤80% amino acid identity with proteins in NCBI database are shown in blue. Scale bar = 0.2 changes/site.

Ampicillin screens led to the identification of 15 unique beta-lactamase genes from all four Amber molecular classes (A–D) (Bush, 2017) (Supplementary Table 1B). The majority of these genes (11) were known beta-lactamase genes (Supplementary Figure 3; Supplementary Table 2) originating mostly from industrial effluent and receiving stream sediment. Three known genes (AMP2_S_DS_C2, AMP3_S_DS_C2, AMP10_S_DS_C2) cluster with the beta-lactamase genes *bla*GES-1 and *bla*VEB-9 (class A) from *K. pneumoniae* and *P. aeruginosa* (Figure 3B), and *bla*CMY-10 (class C) from *A. baumannii* (Supplementary Table 2), all of which are clinically relevant gene families (Paterson and Bonomo, 2005; Jacoby, 2009). In addition to high-level resistance to AMP (MIC > 1,024 mg L^−1^), these enzymes displayed activity against CTX, a 3rd generation cephalosporin (MIC = 8–32 mg L^−1^, Supplementary Table 2). Moreover, the *bla*GES-1 variant is known to display activity against carbapenems, a class of last resort antibiotics (Stewart et al., 2015). All of these observations indicate that the studied effluent-impacted sediment can act as a reservoir of pathogen-borne extended-spectrum beta-lactamases such as the GES, VEB, and CMY-10 types. The detection of these genes in sediment only at the discharge site suggests their accumulation in the environment because of effluent discharge. As effluent from Industry area 2 is mixed with human sewage within the industry, we suspect that these genes could have derived from human sources. Recently, Marathe et al. (2017) showed that untreated urban waste enriches river sediment with GES-type carbapenemases.

Nine remaining known genes cluster with either class D OXA-type genes from *K. pneumoniae* and *A. baumannii* (AMP6_F_WW_C2, AMP18_F_WW_C2, AMP7_S_DS_C2, AMP9_S_DS, AMP18_S_DS_C2, AMP6_S_US_C2, AMP7_F_WW_C2,) or subclass B1-metallo beta-lactamases from *Flavobacterium* sp. (AMP11_F_WW_C2, AMP12_S_DS_C2) (Figures 3A,C). Some of the class D, OXA-type beta-lactamase genes (*bla*OXA-10 and *bla*OXA-198) were obtained from upstream sediment suggesting that the occurrence of these genes was not limited to the release of industrial effluents. As the studied stream flows through the rural area and might be impacted by livestock fecal runoff, the source of the observed *bla*OXA genes is likely attributed to fecal pollution, rather than antibiotic selection pressure from effluent, although the latter cannot be excluded (Agga et al., 2015).

Along with the known genes, four potentially novel beta-lactamases were identified encoding putative class A beta-lactamases (AMP4_S_DS_C2; Figure 3B), two class D beta-lactamases (AMP6_S_DS_C2, AMP1_S_US_C2; Figure 3A) and one class B beta-lactamase (AMP8_S_DS_C2; Figure 3C), sharing 55–74% amino acid sequence identity with known enzymes (Supplementary Table 2). All clones with these genes conferred high-level resistance to AMP (MIC > 1,024 mg L^−1^) and two of them, with class A and class B beta-lactamases (AMP4_S_DS_C2, AMP8_S_DS_C2), conferred additional resistance to CTX (MIC = 8 mg L^−1^, >16 mg L^−1^), suggesting their increased spectrum of activity. Selection on CTX resulted in the identification of a single, known, AmpC beta-lactamase gene originating from discharge sediment (Supplementary Table 2). This gene displayed a high sequence similarity to the *bla*MOX-9 gene from carbapenem-hydrolyzing *Citrobacter freundii*, isolated from a hospital wastewater plant in central Italy (Antonelli et al., 2015). This suggests that the *bla*MOX-9 gene may have originated from human bacteria present in industrial effluent.

Collectively, these results provide a survey of those ARGs in effluents and sediments that are accessible by functional metagenomics. It is also likely that these matrices contain resistance determinants that are not expressed in *E. coli*. Nevertheless, our findings indicate that sediments impacted by antibiotic polluted pharmaceutical effluents could be important sources of clinically relevant known and novel resistance genes, including those conferring resistance to antibiotics that are critically important for human medicine, such as penicillins, 3rd generation cephalosporins, and carbapenems (WHO, 2017).

### Organization and mobility of identified ARGs

**Figure 4:**
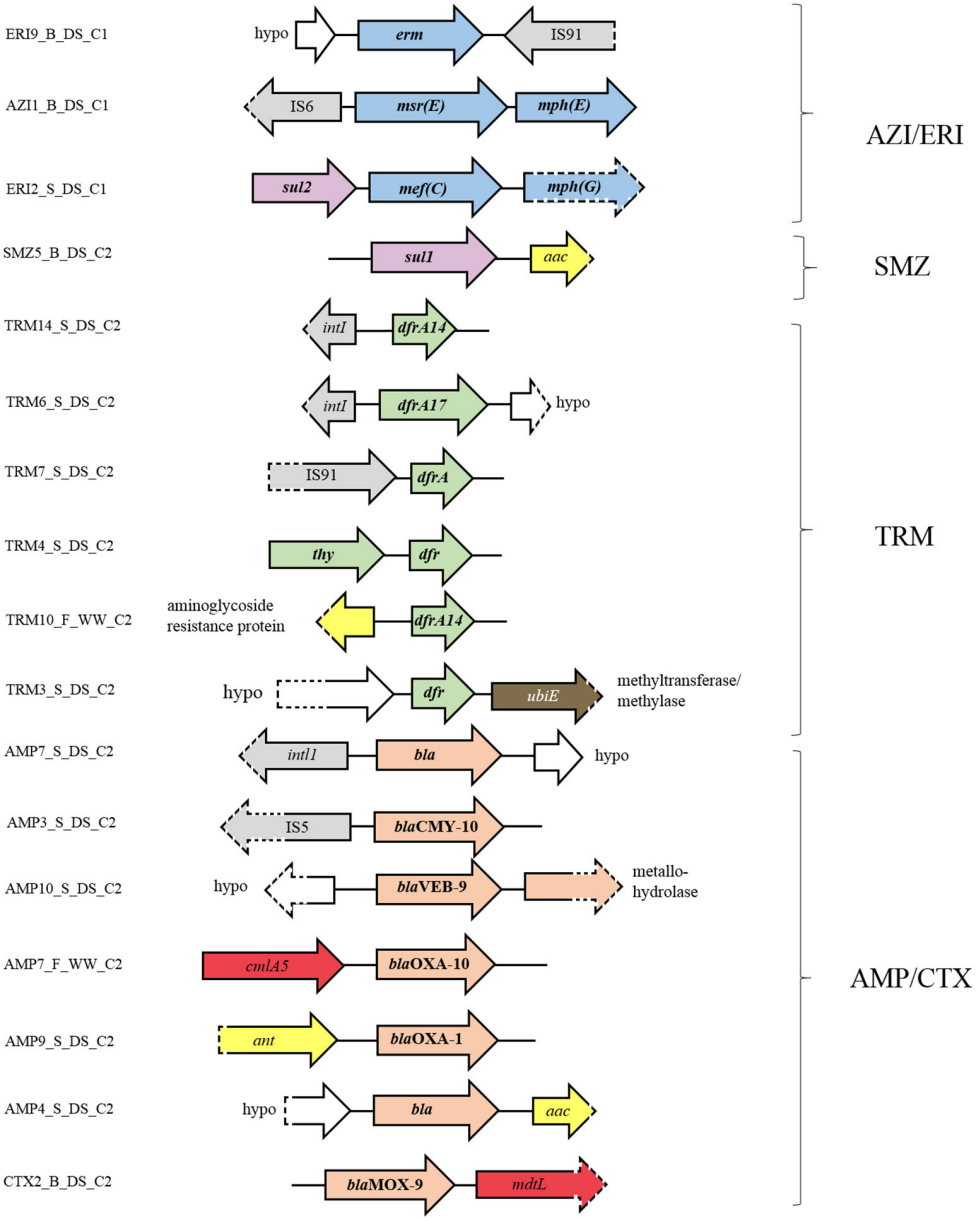
Genetic context of the resistance genes and flanking ORFs identified in the metagenomic libraries. Orientation of the annotated genes in comparison to their genetic context is given by the direction of the arrow. ORFs involved in antibiotic resistance to macrolides are shaded in blue, sulfonamides in purple, trimethoprim in green, beta-lactams in pink, aminoglycosides in yellow, chloramphenicol in red, and D-cycloserine in brown. ORFs connected to gene dissemination are shaded in gray and ORFs annotated as hypothetical proteins in white. Dashed parts of arrows indicate incomplete sequences.

To assess the genomic context of identified ARGs, we studied the available flanking DNA in more detail. Identification of ORFs in macrolide resistant clones revealed that many clones from the libraries of effluent and receiving sediment carried more than one macrolide resistance mechanisms, sometimes on the same mobile element (Figure 4). For example, clone AZI1_B_DS_C1 contained a cluster comprised of genes that encode for a ribosomal protection protein, [*msr*(E)] and a macrolide phosphotransferase [*mph*(E)], separated just by a 55 bp spacer. As observed here, this gene cluster was previously found to be flanked by an IS6 family transposase and is localized on plasmids in different hosts (Schlüter et al., 2007b; Kadlec et al., 2011; Zhang et al., 2013), suggesting that these vectors may play an important role in the dissemination of the *msr*(E)-*mph*(E) cassette. Clone ERI2_S_DS_C1 contained a similar gene cassette composed of the genes *mph*(G) and *mef* (C), which encode a macrolide phosphotransferase and a macrolide efflux pump, respectively. This gene cluster has been found on plasmids from different hosts in Asia (Nonaka et al., 2012; Sugimoto et al., 2017), suggesting its potential for dissemination across species. This seems to be the first time that this gene cassette is reported in Europe. Each gene cluster [*mef* (C)-*mph*(G) and *msr*(E)-*mph*(E)] might be collectively involved in a phenotype of observed high-level resistance to ERI (MIC ≥ 1,024 mg L^−1^) and AZI (MIC ≥ 64 mg L^−1^) as has been previously shown (Schlüter et al., 2007a; Nonaka et al., 2015). The fact that clones with identical gene clusters, either *msr*(E)-*mph*(E) or *mef* (C)-*mph*(G) were found in libraries of effluent and receiving river sediment (Supplementary Figure 5), indicates that industrial effluent is a point source of these gene clusters in river sediment.

Besides carrying genes for two different macrolide resistance mechanisms, some macrolide resistant clones (i.e., clone ERI2_S_DS_C1) carried additional genes, such as *sul2* (sulfonamide resistance). This suggests the potential for co-selection of macrolide and sulfonamide resistance genes as well as their co-transfer under the selection pressure of macrolides and/or other factors.

In addition to clones harboring macrolide resistance genes and deriving from Industry area 1, clones deriving from Industry area 2 and conferring resistance to TRM, sulfonamides and beta-lactams also harbored clusters of ARGs (Figure 4). For example, the TRM resistant clone TRM4_S_DS_C2 contained a cluster comprised of two different genes involved in TRM resistance, *thy* and *dfr*, which have previously been found in bacteria (Kehrenberg and Schwarz, 2005). Clones TRM10_F_WW_C_2 and TRM3_S_DS_C2 harbored clusters containing genes involved in TRM resistance (*dfr14*) and aminoglycoside resistance or TRM resistance (*dfr*) and ubiquinone biosynthesis (methyltransferase). Little is known about the involvement of this methyltransferase in bacterial resistance to antibiotics, though Baisa et al. (2013) reported that the deletion of the *ubiE* gene led to bacterial insensitivity to D-cycloserine, a second-line drug in the treatment of MDR *Mycobacterium tuberculosis* infections. The SMZ resistant clone, SMZ5_B_DS_C2, harbored clustered genes encoding sulfonamide (*sul1*) and aminoglycoside resistance (*aac*). Similarly, genes encoding beta-lactamases were usually clustered with aminoglycoside resistance genes (*ant* or *aac*) or chloramphenicol resistance genes (*cmlA5* or *mdtL*), or co-localized with other beta-lactamase genes (*bla*VEB and metallo hydrolase gene). Finally, we also found that many resistance genes are flanked by mobile genetic elements such as insertion sequence (IS) elements (e.g., IS91, IS5, and IS6) and integron elements (e.g., *int*L and *int*I1 integrase genes).

Taken together, our results and previous studies of sediments subjected to industrial pollution (Kristiansson et al., 2011; Bengtsson-Palme et al., 2014) suggest that ARGs selected in such settings are candidates for dissemination to other bacteria in the environment, including pathogens. Further quantitative studies are needed to assess the transfer of identified genes to other environmental reservoirs or clinical settings. Such studies will provide the basis for future mitigation efforts.

## Conclusions

The present study is the first effort to catalog the resistome from antibiotic polluted pharmaceutical effluents and receiving sediments using functional metagenomics. We highlight these polluted matrices as an important sources of diverse functional ARGs, both known and novel. The association of many of these resistance genes with mobile genetic elements raises the concern that they may spread among bacteria with the potential to reach human pathogens and ultimately lead to clinical failure. Today's traveling habits and trade practices can cause a quick and worldwide spread of any of these resistant bacteria (Zhu et al., 2017). It is of utmost importance to set discharge limits for antibiotics and antibiotic-resistant bacteria from manufacturing sites, in order to limit further evolution of antibiotic resistance in pathogens or commensal bacteria. Furthermore, we need global metagenomic surveys of resistance within high risk habitats such as these impacted by pharmaceutical waste as a prerequisite for proper risk assessment and future mitigation efforts.

## Author contributions

NU-K: Designed the research; NU-K, JG-P, MM, AŠ, and AB: Collected the samples; JG-P, MM, AŠ, and AB: Performed the experiments; NU-K, JG-P, AŠ, MM, and FW: Analyzed the data and prepared the figures; NU-K, JG-P, and AŠ: Wrote the paper. All authors read and approved the final manuscript.

### Conflict of interest statement

The authors declare that the research was conducted in the absence of any commercial or financial relationships that could be construed as a potential conflict of interest.
