# Corrigendum: Functional Repertoire of Antibiotic Resistance Genes in Antibiotic Manufacturing Effluents and Receiving Freshwater Sediments

**DOI:** 10.3389/fmicb.2018.02502

**Published:** 2018-10-16

**Authors:** Juan J. González-Plaza, Ana Šimatović, Milena Milaković, Ana Bielen, Fabienne Wichmann, Nikolina Udiković-Kolić

**Affiliations:** ^1^Division for Marine and Environmental Research, Ruder Bošković Institute, Zagreb, Croatia; ^2^Division of Molecular Biology, Ruder Bošković Institute, Zagreb, Croatia; ^3^Department of Biochemical Engineering, Faculty of Food Technology and Biotechnology, University of Zagreb, Zagreb, Croatia; ^4^Independent Researcher, Lausanne, Switzerland

**Keywords:** antibiotic resistance, effluent, manufacturing, antibiotic pollution, sediment, macrolides, functional metagenomics

In the original article, the reference for Sugimoto et al. (2017) was incorrectly written as Sugimoto, A., Maeda, A., Itto, K., and Arimoto, H. (2017). Deciphering the mode of action of cell wall-inhibiting antibiotics using metabolic labeling of growing peptidoglycan in *Streptococcus pyogenes*. *Sci. Rep*. 7:1129. doi: 10.1038/s41598-017-01267-5. The correct reference appears in the Reference List below. The authors apologize for this error and state that this does not change the scientific conclusions of the article in any way.

The original article has been updated.

## Conflict of interest statement

The authors declare that the research was conducted in the absence of any commercial or financial relationships that could be construed as a potential conflict of interest.

## References

[B1] Sugimoto Y. Suzuki S. Nonaka L. Boonla C. Sukpanyatham N. Chou H.-Y. Wu J.-H. (2017). The novel *mef* (C)-*mph*(G) macrolide resistance genes are conveyed in the environment on various vectors. J. Glob. Antimicrob. Resist. 10, 47–53. 10.1016/j.jgar.2017.03.015 28689921

