# Supplementary material for: Functional Repertoire of Antibiotic Resistance Genes in Antibiotic Manufacturing Effluents and Receiving Freshwater Sediments
Source: Front Microbiol. 2018 Jan 17;8:2675. doi: 10.3389/fmicb.2017.02675 (PMC5776109; doi:10.3389/fmicb.2017.02675)
Supplement: Supplementary file 1 [file DataSheet1.DOCX]

Supplementary Material

Functional repertoire of antibiotic resistance genes in antibiotic manufacturing effluents and receiving freshwater sediments

Juan José González-Plaza*, Ana Šimatović*, Milena Milaković, Ana Bielen, Fabienne Wichmann, Nikolina Udiković-Kolić^**^

* Equal contribution

** Corresponding author: Ruđer Bošković Institute, Division for Marine and Environmental Research, Bijenička 54, P.O. Box 180; 10002 Zagreb, Croatia.

Phone:[+385-1-4680-944](tel:%2B385-1-4680-944)

E-mail address: [nudikov@irb.hr](mailto:nudikov@irb.hr)

# Supplementary Figures and Tables

## Supplementary Figures

##
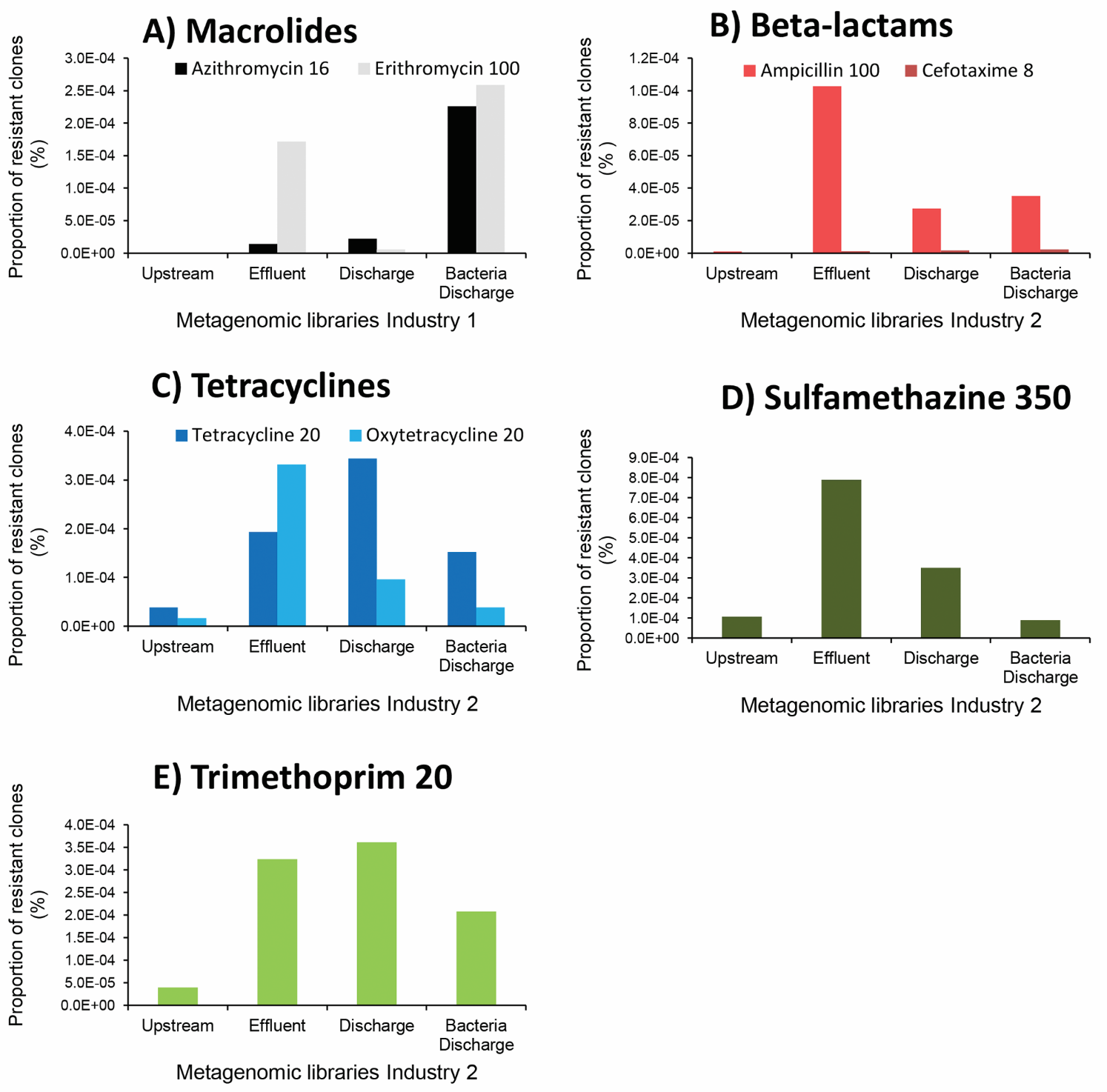


**Supplementary Figure 1.** Proportion of clones resistant to eight different antibiotics, obtained in functional metagenomics libraries from effluent and sediments of Industry area 1 (**A**) and Industry area 2 (**B**, **C**, **D**, **E**, and **F**). Antibiotics used for the screening are indicated above each plot area, and the accompanying number indicates the final antibiotic concentration (mg L^-1^). The proportion of antibiotic-resistant clones in each library was calculated as the ratio of the number of resistant clones and total number of clones.

**
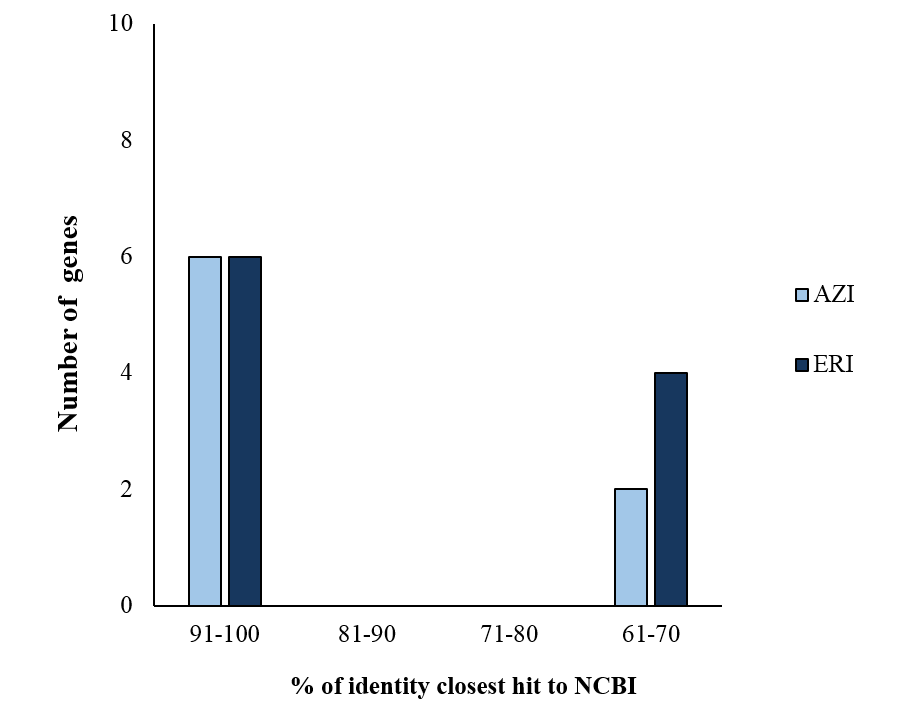
**

**Supplementary Figure 2.** Distribution of amino acid identity of azithromycin (AZI) and erythromycin (ERI) resistance genes from Industry area 1 to homologues in the NCBI database.


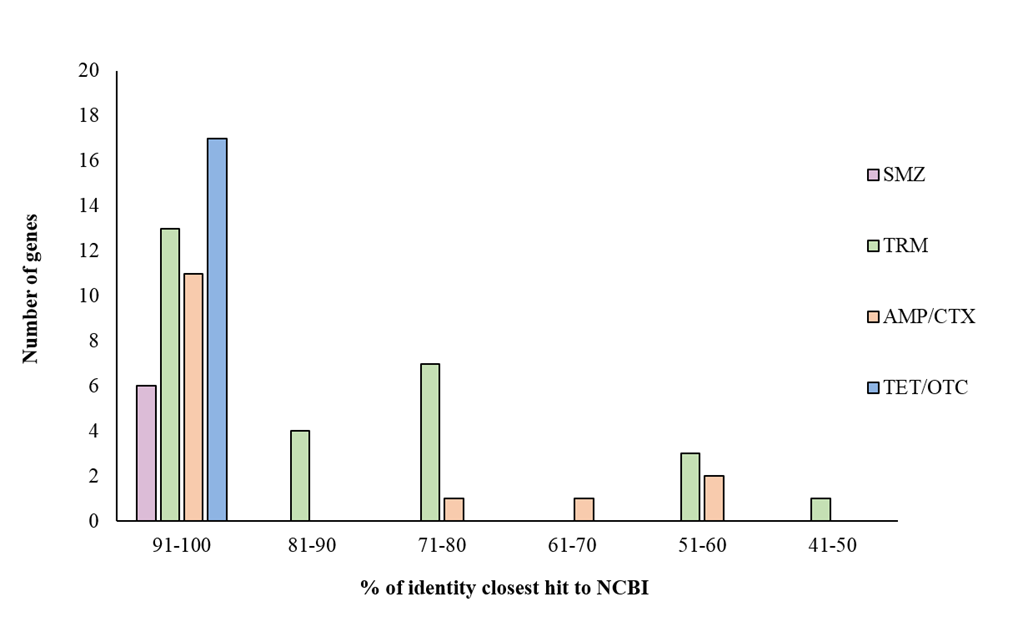
**Supplementary figure 3.** Distribution of amino acid identity of unique antibiotic resistance genes from Industry area 2 to homologues in the NCBI database. SMZ-sulfamethazine, TRM-trimethoprim, AMP-ampicillin, CTX-cefotaxime, TET-tetracycline, OTC-oxytetracycline.

**
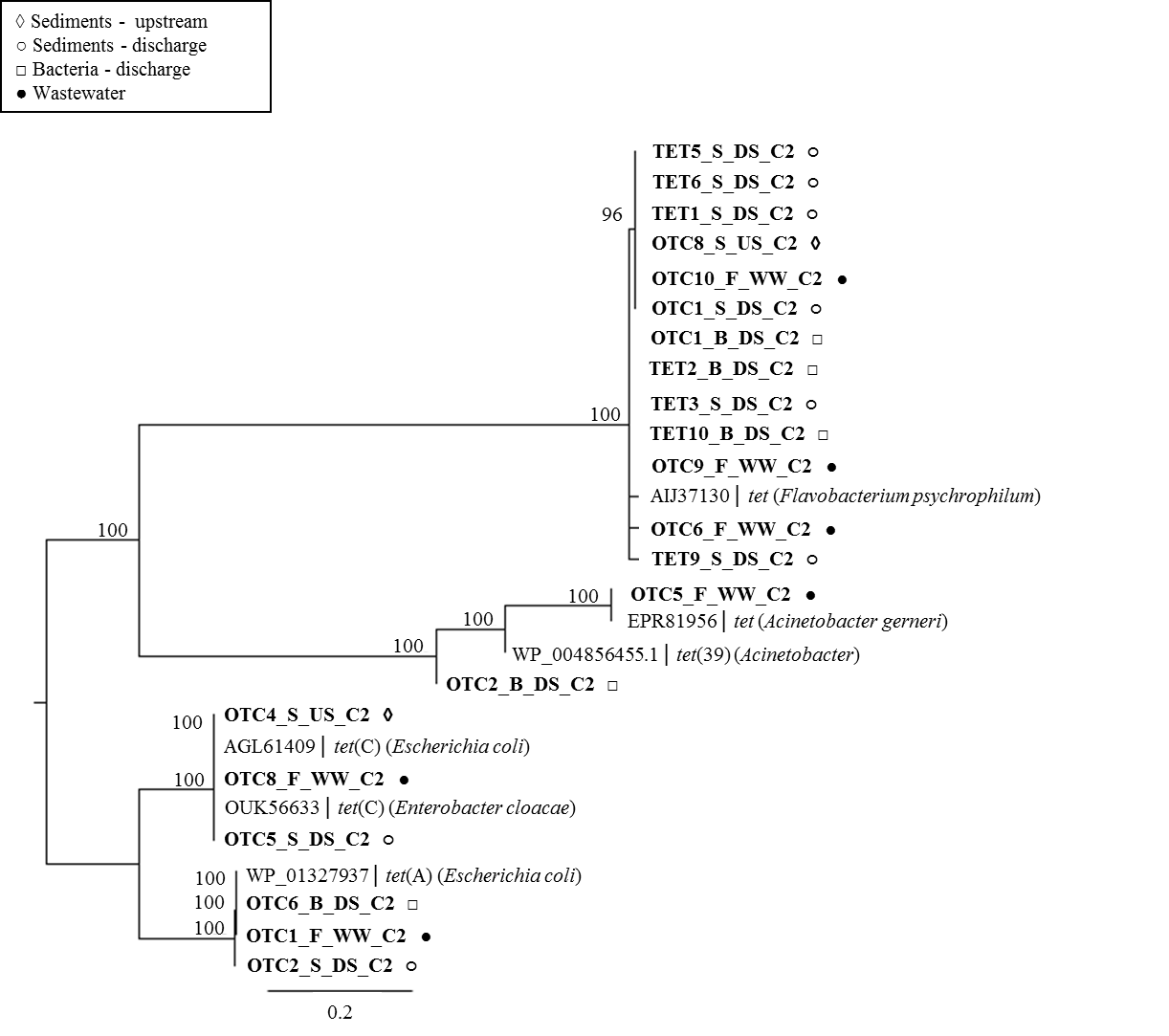
**

**Supplementary Figure 4.** Phylogenetic tree of protein sequences of tetracycline (TET) and oxytetracycline (OTC) resistance genes. Best BLAST hits and representative protein sequences of the studied genes were retrieved from the NCBI database. The evolutionary history was inferred by using the maximum likelihood method and the Geneious software. Bootstrap values were calculated on 100 replications and only those higher than 80% are shown. Scale bar=0.2 changes/site.


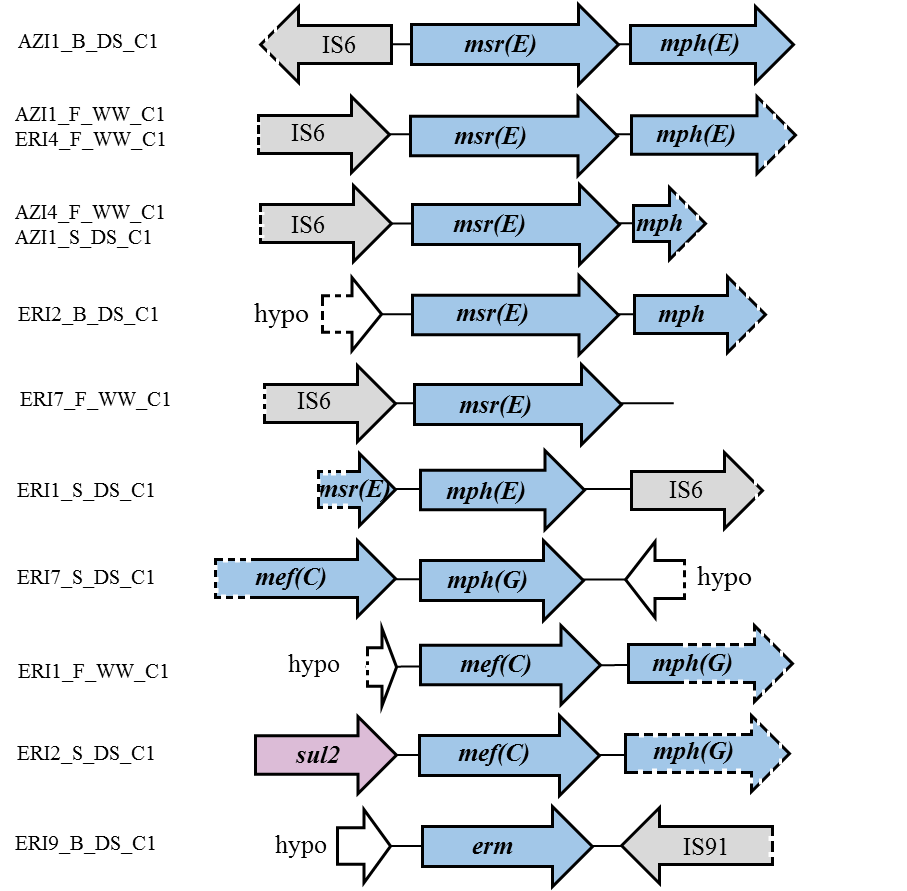


**Supplementary Figure 5.** Genetic contexts of the macrolide resistance genes identified in the metagenomic libraries from Industry area 1. ORFs are represented by arrows, which are shaded in blue for ORFs involved in antibiotic resistance towards macrolides, in purple for ORF involved in antibiotic resistance towards sulfonamides, in grey for ORFs related to gene dissemination and in white for ORFs annotated as hypothetical proteins. Dashed parts of arrows indicate incomplete sequences.

## Supplementary Tables

**Supplementary Table 1.** Number of unique ARGs detected in the libraries from Industry area 1 (**A**) and Industry area 2 (**B**). The detected resistance genes were classified based on the mechanism of antibiotic resistance.

**A)**

| Industry area 1 | | |
| --- | --- | --- |
| Library | **Mechanism of macrolide resistance** | **Number of unique ARGS** |
| S_US_C1 | Unknown- GTPase binding protein | **4** |
| F_WW_C1 | Efflux pump | **1** |
|  | Ribosomal protection | **3** |
| B_DS_C1 | Efflux pump | **1** |
|  | Macrolide inactivation | **1** |
|  | Methylation of rRNA | **1** |
|  | Ribosomal protection | **1** |
| S_DS_C1 | Efflux pump | **1** |
|  | Macrolide inactivation | **2** |
|  | Ribosomal protection | **1** |
| TOTAL: | | **16** |

**B)**

| Industry area 2 | | |
| --- | --- | --- |
| Library | **Mechanism of antibiotic resistance** | **Number of unique ARGs** |
| S_US_C2 | Modification of target site - dihydropteroate synthase | **1** |
|  | Modification of target site - dihydrofolate reductase | **4** |
|  | Modification of target site - thymidylate synthase | **3** |
|  | Efflux - tetracycline transporter | **2** |
|  | Antibiotic inactivation - beta-lactamase | **2** |
| F_WW_C2 | Modification of target site - dihydropteroate synthase | **1** |
|  | Modification of target site - dihydrofolate reductase | **5** |
|  | Modification of target site - thymidylate synthase | **1** |
|  | Efflux - tetracycline transporter | **6** |
|  | Antibiotic inactivation - beta-lactamase | **3** |
| B_DS_C2 | Modification of target site - dihydropteroate synthase | **2** |
|  | Modification of target site - dihydrofolate reductase | **3** |
|  | Efflux - tetracycline transporter | **4** |
|  | Antibiotic inactivation - beta-lactamase | **1** |
| S_DS_C2 | Modification of target site - dihydropteroate synthase | **2** |
|  | Modification of target site - dihydrofolate reductase | **11** |
|  | Modification of target site - thymidylate synthase | **1** |
|  | Efflux - tetracycline transporter | **5** |
|  | Antibiotic inactivation - beta-lactamase | **9** |
| TOTAL: | | **66** |

**Supplementary Table 2.** Resistance genes identified in metagenomic clones with distinct restriction patterns, from libraries of Industry area 2. Clones containing inserts conferring resistance towards sulfamethazine (SMZ), trimethoprim (TRM), oxytetracycline (OTC), tetracycline (TET), ampicillin (AMP) or cefotaxime (CTX) are shown. MIC: Minimum Inhibitory Concentration. Unique genes (based on their nucleotide sequence) from the same library are marked with *.

| Antibiotic used for selection | Clone designation / origin | MIC  (mg L^−1^) | Gene length (bp) | Gene annotation (Closest BLASTX hit in NCBI) | % amino acid identity. | GenBank Accession # |
| --- | --- | --- | --- | --- | --- | --- |
| Sulfamethazine | SMZ2_S_US_C2/  Upstream sediment | >1 024 (SMZ) | 927 | Dihydropteroate synthase Sul1  (*Enterobacter cloacae* WP_012695459.1) * | 99 | MG585961 |
|  | SMZ7_S_US_C2/  Upstream sediment | >1 024 (SMZ) | 927 | Dihydropteroate synthase Sul1  (*Enterobacter cloacae* WP_012695459.1) | 99 | MG585962 |
|  | SMZ10_S_US_C2/  Upstream sediment | >1 024 (SMZ) | 927 | Dihydropteroate synthase Sul1  (*Enterobacter cloacae* WP_012695459.1) | 99 | MG585963 |
|  | SMZ5_F_WW_C2/  Effluent | >1 024 (SMZ) | 927 | Dihydropteroate synthase Sul1  (*Enterobacter cloacae* WP_012695459.1) * | 99 | MG585964 |
|  | SMZ6_F_WW_C2/  Effluent | >1 024 (SMZ) | 927 | Dihydropteroate synthase Sul1  (*Enterobacter cloacae* WP_012695459.1) | 99 | MG585965 |
|  | SMZ10_F_WW_C2/  Effluent | >1 024 (SMZ) | 927 | Dihydropteroate synthase Sul1  (*Enterobacter cloacae* WP_012695459.1) | 99 | MG585966 |
|  | SMZ1_B_DS_C2/  Sediment bacteria | >1 024 (SMZ) | 852 | Dihydropteroate synthase Sul2  (*Escherichia coli* HVH 213 EQU75297.1) * | 99 | MG585967 |
|  | SMZ5_B_DS_C2/  Sediment bacteria | >1 024 (SMZ) | 927 | Dihydropteroate synthase Sul1  (*Enterobacter cloacae* WP_012695459.1) * | 99 | MG585968 |
|  | SMZ9_B_DS_C2/  Sediment bacteria | >1 024 (SMZ) | 927 | Dihydropteroate synthase Sul1  (*Enterobacter cloacae* WP_012695459.1) | 99 | MG585969 |
|  | SMZ6_S_DS_C2/ Discharge sediment | >1 024 (SMZ) | 927 | Dihydropteroate synthase Sul1  (*Acinetobacter baumannii* ADX02776.1) * | 99 | MG585970 |
|  | SMZ8_S_DS_C2/ Discharge sediment | >1 024 (SMZ) | 927 | Dihydropteroate synthase Sul1  (*Enterobacter cloacae* WP_012695459.1) * | 99 | MG585971 |
| Trimethoprim | TRM2_S_US_C2/ Upstream sediment | 512 (TRM) | 795 | Thymidylate synthase  (*Bacteroidetes bacterium* GWB2_41_8 OFX59224) * | 90 | MG585972 |
|  | TRM5_S_US_C2/ Upstream sediment | >512 (TRM) | 795 | Thymidylate synthase  (*Neisseria* sp. HMSC15C08 WP_070491626.1) * | 80 | MG585973 |
|  | TRM6_S_US_C2/ Upstream sediment | >512 (TRM) | 504 | Dihydrofolate reductase  (*Nitrosomonas nitrosa* SFM09632.1) * | 53 | MG585975 |
|  | TRM7_S_US_C2/ Upstream sediment | >512 (TRM) | 483 | Dihydrofolate reductase  (*Methylotenera versatilis* WP_047542178.1)* | 74 | MG585976 |
|  | TRM8_S_US_C2/ Upstream sediment | >512 (TRM) | 528 | Dihydrofolate reductase  (*Lactonifactor longoviformis* WP_072849072.1) * | 49 | MG585977 |
|  | TRM10_S_US_C2/ Upstream sediment | >512 (TRM) | 498 | Dihydrofolate reductase  (*Lentimicrobium saccharophilum* WP_062039092.1) * | 57 | MG585978 |
|  |  |  | 795 | Thymidylate synthase  (*Bacteroidetes bacterium* GWA2_40_15 OFX42999.1) * | 80 | MG585974 |
|  | TRM2_F_WW_C2/  Effluent | >512 (TRM) | 483 | Dihydrofolate reductase DfrA14  (*Escherichia coli* YP_006953259.1) * | 100 | MG585979 |
|  | TRM6_F_WW_C2/  Effluent | >512 (TRM) | 294 | Dihydrofolate reductase DHFR2  (*Pseudomonas aeruginosa* CAQ52800.1) * | 99 | MG585986 |
|  | TRM8_F_WW_C2/  Effluent | >512 (TRM) | 483 | Dihydrofolate reductase DfrA14  (*Salmonella enterica* NP_569370.1) * | 100 | MG585980 |
|  | TRM10_F_WW_C2/  Effluent | >512 (TRM) | 483 | Dihydrofolate reductase DfrA14  (*Salmonella enterica* NP_569370.1) * | 99 | MG585981 |
|  | TRM13_F_WW_C2/  Effluent | >512 (TRM) | 483 | Dihydrofolate reductase DfrA14  (*Escherichia coli* YP_006953259.1) | 100 | MG585982 |
|  | TRM16_F_WW_C2/  Effluent | >512 (TRM) | 486 | Dihydrofolate reductase  (*Flavobacterium sasangense* WP_026725269.1) * | 93 | MG585985 |
|  | TRM17_F_WW_C2/  Effluent | >512 (TRM) | 483 | Dihydrofolate reductase DfrA14  (*Salmonella enterica* NP_569370.1) | 100 | MG585983 |
|  | TRM19_F_WW_C2/  Effluent | >512 (TRM) | 843 | Thymidylate synthase  (*Acinetobacter lwoffii* WP_004729505.1) * | 100 | MG585984 |
|  | TRM1_B_DS_C2/  Sediment bacteria | >512 (TRM) | 483 | Dihydrofolate reductase DfrA14  (*Escherichia coli* YP_006953259.1) * | 100 | MG585987 |
|  | TRM2_B_DS_C2/  Sediment bacteria | >512 (TRM) | 483 | Dihydrofolate reductase DfrA14  (*Escherichia coli* YP_006953259.1) | 100 | MG585988 |
|  | TRM4_B_DS_C2/  Sediment bacteria | >512 (TRM) | 483 | Dihydrofolate reductase DfrA14  (*Escherichia coli* YP_006953259.1) | 100 | MG585989 |
|  | TRM5_B_DS_C2/  Sediment bacteria | >512 (TRM) | 486 | Dihydrofolate reductase  (*Neisseria wadsworthii* WP_040666967.1) * | 78 | MG585992 |
|  | TRM6_B_DS_C2/  Sediment bacteria | >512 (TRM) | 459 | Dihydrofolate reductase DfrA12  (*Escherichia coli* WP_071846383.1) * | 100 | MG585991 |
|  | TRM7_B_DS_C2/  Sediment bacteria | >512 (TRM) | 483 | Dihydrofolate reductase DfrA14  (*Escherichia coli* YP_006953259.1) | 100 | MG585990 |
|  | TRM2_S_DS_C2/ Discharge sediment | 512 (TRM) | 483 | Dihydrofolate reductase  (*Flavobacterium sinopsychrotolerans* WP_091173155.1) * | 84 | MG585993 |
|  | TRM3_S_DS_C2/ Discharge sediment | >512 (TRM) | 486 | Dihydrofolate reductase  (*Flavobacterium sasangense* WP_026725269.1) * | 94 | MG585994 |
|  | TRM4_S_DS_C2/ Discharge sediment | >512 (TRM) | 510 | Dihydrofolate reductase  (*Ruminococcaceae bacterium* YRB3002 SCW70680.1) * | 59 | MG585995 |
|  | TRM5_S_DS_C2/ Discharge sediment | >512 (TRM) | 795 | Thymidylate synthase  (*Bdellovibrio exovorus* WP_015470741.1) * | 87 | MG586006 |
|  |  |  | 507 | Dihydrofolate reductase  (uncultured bacteria AIA18652.1) * | 72 | MG585996 |
|  | TRM6_S_DS_C2/ Discharge sediment | >512 (TRM) | 702 | Dihydrofolate reductase DfrA17  (*Klebsiella pneumoniae* WP_013023850.1) * | 93 | MG586003 |
|  | TRM7_S_DS_C2/ Discharge sediment | >512 (TRM) | 477 | Dihydrofolate reductase DfrA1  (*Citrobacter freundii* WP_053764311.1) * | 99 | MG586002 |
|  | TRM8_S_DS_C2/ Discharge sediment | 512 (TRM) | 486 | Dihydrofolate reductase  (*Flavobacterium sasangense* WP_026725269.1) | 94 | MG585997 |
|  | TRM9_S_DS_C2/ Discharge sediment | >512 (TRM) | 483 | Dihydrofolate reductase DfrA14  (*Escherichia coli* YP_006953259.1) * | 100 | MG586004 |
|  | TRM10_S_DS_C2/ Discharge sediment | >512 (TRM) | 498 | Dihydrofolate reductase  (*Paludibacter propionicigenes* WP_013444380) * | 75 | MG585998 |
|  | TRM11_S_DS_C2/ Discharge sediment | 512 (TRM) | 483 | Dihydrofolate reductase  (*Flavobacterium sinopsychrotolerans* WP_091173155.1) | 84 | MG585999 |
|  | TRM12_S_DS_C2/ Discharge sediment | >512 (TRM) | 483 | Dihydrofolate reductase  (*Flavobacterium sinopsychrotolerans* WP_091173155.1) * | 84 | MG586000 |
|  | TRM14_S_DS_C2/ Discharge sediment | >512 (TRM) | 483 | Dihydrofolate reductase DfrA14  (*Salmonella enterica* NP_569370.1) * | 100 | MG586005 |
|  | TRM19_S_DS_C2/ Discharge sediment | >512 (TRM) | 495 | Dihydrofolate reductase  (*Flavobacterium antarcticum* WP_022826597.1) * | 79 | MG586001 |
| Oxytetracycline | OTC4_S_US_C2/  Upstream sediment | 128 (OTC)  64 (TET) | 1 191 | Tetracycline MFS efflux pump  (*Escherichia coli* AGL61409) * | 100 | MG586007 |
|  | OTC8_S_US_C2/  Upstream sediment | 128 (OTC)  128 (TET) | 1 320 | Tetracycline MFS efflux pump  (*Flavobacterium psychrophilum*  AIJ37130.1) * | 97 | MG586008 |
|  | OTC1_F_WW_C2/  Effluent | 512 (OTC) 256 (TET) | 1 275 | Tetracycline MFS efflux pump  (*Escherichia coli* WP_01327937.1) * | 100 | MG586009 |
|  | OTC5_F_WW_C2/  Effluent | 128 (OTC) 128 (TET) | 1 122 | Tetracycline MFS efflux pump  (*Acinetobacter gerneri* EPR81956.1 ) * | 100 | MG586011 |
|  | OTC6_F_WW_C2/  Effluent | 128 (OTC) 128 (TET) | 1 317 | Tetracycline efflux pump  (*Flavobacterium psychrophilum*  AIJ37130.1) * | 94 | MG586012 |
|  | OTC8_F_WW_C2/  Effluent | 128 (OTC) 64 (TET) | 1 191 | Tetracycline MFS efflux pump  (*Enterobacter cloacae* OUK56633.1) * | 100 | MG586010 |
|  | OTC9_F_WW_C2/  Effluent | 128 (OTC) 128 (TET) | 1 320 | Tetracycline MFS efflux pump  (*Flavobacterium psychrophilum*  AIJ37130.1) * | 99 | MG586013 |
|  | OTC10_F_WW_C2/  Effluent | 64 (OTC) 64 (TET) | 1 320 | Tetracycline MFS efflux pump  (*Flavobacterium psychrophilum*  AIJ37130.1) * | 97 | MG586014 |
|  | OTC1_B_DS_C2/  Sediment bacteria | 512 (OTC)  128 (TET) | 1 320 | Tetracycline MFS efflux pump  (*Flavobacterium psychrophilum*  AIJ37130.1) * | 99 | MG586029 |
|  | OTC2_B_DS_C2/  Sediment bacteria | 128 (OTC)  128 (TET) | 1 188 | Tetracycline MFS efflux pump  (*Acinetobacter* WP_004856455) * | 99 | MG586033 |
|  | OTC6_B_DS_C2/  Sediment bacteria | 128 (OTC)  64 (TET) | 1 275 | Tetracycline MFS efflux pump  (*Escherichia coli* WP_01327937.1) * | 100 | MG586032 |
|  | OTC1_S_DS_C2/ Discharge sediment | 128 (OTC)  128 (TET) | 1 320 | Tetracycline MFS efflux pump  (*Flavobacterium psychrophilum*  AIJ37130.1) * | 97 | MG586015 |
|  | OTC2_S_DS_C2/ Discharge sediment | 128 (OTC)  128 (TET) | 1 275 | Tetracycline MFS efflux pump  (*Escherichia coli* WP_01327937.1) * | 100 | MG586022 |
|  | OTC5_S_DS_C2/ Discharge sediment | 64 (OTC)  64 (TET) | 1 191 | Tetracycline MFS efflux pump  (*Escherichia coli* AGL61409) * | 100 | MG586021 |
| Tetracycline | TET2_B_DS_C2/  Sediment bacteria | 64 (TET)  64 (OTC) | 1 320 | Tetracycline MFS efflux pump  (*Flavobacterium psychrophilum* AIJ37130.1) | 99 | MG586030 |
|  | TET10_B_DS_C2/  Sediment bacteria | 64 (TET)  64 (OTC) | 1 320 | Tetracycline MFS efflux pump  (*Flavobacterium psychrophilum*  AIJ37130.1) * | 98 | MG586031 |
|  | TET1_S_DS_C2/ Discharge sediment | 64 (TET)  64 (OTC) | 1 320 | Tetracycline MFS efflux pump  (*Flavobacterium psychrophilum* AIJ37130.1) | 97 | MG586016 |
|  | TET3_S_DS_C2/ Discharge sediment | 64 (TET)  64 (OTC) | 1 320 | Tetracycline MFS efflux pump  (*Flavobacterium psychrophilum*  AIJ37130.1) * | 99 | MG586017 |
|  | TET5_S_DS_C2/ Discharge sediment | 64 (TET)  64 (OTC) | 1 320 | Tetracycline MFS efflux pump  (*Flavobacterium psychrophilum* AIJ37130.1) | 97 | MG586018 |
|  | TET6_S_DS_C2/ Discharge sediment | 64 (TET)  64 (OTC) | 1 320 | Tetracycline MFS efflux pump  (*Flavobacterium psychrophilum* AIJ37130.1) | 99 | MG586019 |
|  | TET9_S_DS_C2/ Discharge sediment | 64 (TET)  64 (OTC) | 1 320 | Tetracycline MFS efflux pump  (*Flavobacterium psychrophilum*  AIJ37130.1) * | 98 | MG586020 |
| Ampicillin | AMP1_S_US_C2/  Upstream sediment | >1 024 (AMP)  <8 (CTX) | 777 | Class D beta-lactamase OXA-198  (*Pseudomonas aeruginosa*  WP_094865115.1) * | 70 | MG586024 |
|  | AMP6_S_US_C2/  Upstream sediment | >1 024 (AMP)  <8 (CTX) | 828 | Class D beta-lactamase OXA-10  (*Klebsiella pneumoniae*  WP_020442392.1) * | 99 | MG586023 |
|  | AMP6_F_WW_C2/  Effluent | >1 024 (AMP)  <0.25 (CTX) | 849 | Class D beta-lactamase  (uncultured bacterium AMP47957.1) * | 99 | MG586025 |
|  | AMP7_F_WW_C2/  Effluent | >1 024 (AMP)  <0.25 (CTX) | 828 | Class D beta-lactamase OXA-10  (*Klebsiella pneumoniae*  WP_020442392.1) * | 99 | MG586028 |
|  | AMP11_F_WW_C2/  Effluent | >1 024 (AMP)  <0.25 (CTX) | 741 | Subclass B1 metallo-beta-lactamase  (*Flavobacterium plurextorum* WP_089058075) * | 98 | MG586026 |
|  | AMP18_F_WW_C2/  Effluent | >1 024 (AMP)  <0.25 (CTX) | 849 | Class D beta-lactamase  (uncultured bacterium AMP47957.1) | 99 | MG586027 |
|  | AMP2_S_DS_C2/ Discharge sediment | >1 024 (AMP)  >16 (CTX) | 864 | Class A extended-spectrum beta-lactamase GES-1  (*Klebsiella pneumoniae* AAO32356.1) * | 100 | MG586040 |
|  | AMP3_S_DS_C2/ Discharge sediment | >1 024 (AMP) 8 (CTX) | 1 152 | Class C beta-lactamase CMY-10  (*Acinetobacter baumannii* EXB07206.1) * | 99 | MG586041 |
|  | AMP4_S_DS_C2/ Discharge sediment | >1 024 (AMP) >16 (CTX) | 906 | Class A beta-lactamase  (*Pedobacter* sp. PACM 27299 ALL06350.1)* | 59 | MG586035 |
|  | AMP6_S_DS_C2/ Discharge sediment | >1 024 (AMP) <0.25 (CTX) | 786 | Class D beta-lactamase  (*Desulforegula conservatri*x WP_027358149.1) * | 55 | MG586036 |
|  | AMP7_S_DS_C2/ Discharge sediment | >1 024 (AMP) <0.25 (CTX) | 903 | Class D beta-lactamase  (uncultured bacterium AMP47162.1) * | 100 | MG586037 |
|  | AMP8_S_DS_C2/ Discharge sediment | >1 024 (AMP)  8 (CTX) | 855 | BJP beta-lactamase  (uncultured bacterium AIA18864) * | 74 | MG586038 |
|  | AMP9_S_DS_C2/ Discharge sediment | 512 (AMP) <0.25 (CTX) | 876 | Beta-lactamase OXA-1 precursor  (*Acinetobacter baumannii* SCY69726.1) * | 99 | MG586043 |
|  | AMP10_S_DS_C2/ Discharge sediment | >1 024 (AMP) 32 (CTX) | 900 | Class A extended-spectrum beta-lactamase VEB-9  (*Pseudomonas aeruginosa* OOK92124.1) * | 100 | MG586042 |
|  | AMP12_S_DS_C2/ Discharge sediment | >1 024 (AMP)  8 (CTX) | 741 | Subclass B1 metallo-beta-lactamase  (*Flavobacterium oncorhynchi* WP_089052251.1) * | 97 | MG586039 |
|  | AMP18_S_DS_C2/ Discharge sediment | 1 024 (AMP)  >0.25 (CTX) | 876 | Class D beta-lactamase OXA-1  (*Acinetobacter baumannii* SCY69726.1) | 99 | MG586044 |
| Cefotaxime | CTX2_B_DS_C2/  Sediment bacteria | 32 (CTX) | 1 152 | CMY-1/MOX family class C beta-lactamase MOX-9  (*Citrobacter freundii* AIG22447.1) * | 98 | MG586034 |
